# ACUTE RESPIRATORY DISEASES IN BRAZILIAN CHILDREN: ARE CAREGIVERS ABLE TO DETECT EARLY WARNING SIGNS?

**DOI:** 10.1590/1984-0462/;2018;36;1;00008

**Published:** 2018-01-15

**Authors:** Saulo Duarte Passos, Francila Ferreira Maziero, Diego Quilles Antoniassi, Lidiane Trevisan de Souza, Arianna Freire Felix, Eloise Dotta, Monica Ester Orensztejn, Evaldo Marchi, Rosa Estela Gazeta

**Affiliations:** a Faculdade de Medicina de Jundiaí (FMJ), Jundiaí, SP, Brasil.; b Hospital Universitário, FMJ, Jundiaí, SP, Brasil.

**Keywords:** Tachypnea, Respiratory tract infections, Knowledge, Medical care, Pneumonia, Diagnosis, Taquipneia, Infecções do trato respiratório, Conhecimento, Cuidados médicos, Pneumonia, Diagnóstico

## Abstract

**Objective:**

To assess the level of caregiver knowledge about respiratory signs and symptoms of acute respiratory infection (ARI) as well as their ability to detect the early warning signs and need for medical assistance in children referred to an emergency service.

**Methods:**

This is a prospective, cross-sectional study. A standardized questionnaire with questions on the perception of the severity of ARI signs and symptoms was applied to caregivers of pediatric patients assisted in the emergency room of a university hospital from August 2011 to May 2012. Chi-square and Student’s t-tests were used to determine which variables contributed with caregivers’ recognition of severity of acute respiratory diseases.

**Results:**

499 caregivers were interviewed. The most cited causes of ARI were flu syndrome (78.6%), common cold (73.9%), pharyngitis (64.1%), and pneumonia (54.5%). Fever (34.1%) and cough (15.8%) were major reasons for referral to hospital. The most cited signs of severity recognized by caregivers were fever (99.6%), dyspnea (91.4%), wheezing (86.4%), adynamia (80.2%), coughing (79.8%), and tachypnea (78.6%). Children’s history of respiratory diseases (p=0.002), caregiver’s age (p=0.010) and marital status (p=0.014) were significantly associated with tachypnea, the most severe ARI symptom.

**Conclusions:**

Although caregivers of children can recognize ARI most important signs and symptoms, they are unable to judge severity, which may delay medical care and early treatment.

## RESUMO

**Objetivo:**

Avaliar o nível de conhecimento do cuidador em relação aos sinais e sintomas respiratórios de Infecções Respiratórias Agudas (IRA) e a percepção dos mesmos em relação às crianças que necessitam de assistência médica.

**Métodos:**

Estudo prospectivo e transversal, no qual um questionário padronizado com itens relacionados à percepção da gravidade dos sinais e sintomas de IRA foi administrado a cuidadores de pacientes pediátricos admitidos no serviço de emergência de um hospital universitário no período de agosto de 2011 a maio de 2012. A análise estatística foi realizada com os testes do qui-quadrado e t-Student para determinar quais variáveis contribuíram para o reconhecimento pelos cuidadores da gravidade das doenças respiratórias agudas.

**Resultados:**

Foram entrevistados 499 cuidadores. As causas de IRA mais citadas foram Síndrome gripal (78,6%), Resfriado comum (73,9%), Faringites (64,1%) e Pneumonia (54,5%). Febre (34,1%) e Tosse (15,8%) foram as principais razões para a procura de atendimento. Os sinais de gravidade mais citados pelos cuidadores foram: febre (99,6%), dispneia (91,4%), sibilância (86,4%), adinamia (80,2%), tosse (79,8%) e taquipneia (78,6%). O histórico de doença respiratória anterior do paciente (p=0,002), a idade (p=0,010) e o estado civil do cuidador (p=0,014) foram as variáveis significativamente associadas com taquipneia, o sintoma mais grave de IRA.

**Conclusões:**

Embora cuidadores pediátricos possam perceber os principais sinais de IRA, eles não são capazes de reconhecer a gravidade destes, o que pode atrasar os cuidados médicos e impedir o tratamento precoce.

## INTRODUCTION

Pneumonia is a major public health concern in developing countries, particularly among children under 5 years of age. Approximately 150 million new cases of pneumonia occur each year; 11-20 million children require hospitalization, and 2 million die.^1^ In Brazil, respiratory diseases (especially pneumonia) are responsible for 22.3% of all deaths among 1- to 4-year-olds, ranking as the leading cause of death for this age group. Pneumonia is associated with a high rate of hospitalization^2^, and 30% to 50% of children taken to emergency or basic medical care have respiratory symptoms.^3^

Clinical and radiological diagnosis of pneumonia in children can be difficult, although physical and radiological signs are readily recognized in pediatric practice.^4^ In 1980, the World Health Organization (WHO) developed the first guidelines for the diagnosis and management of pneumonia in children in developing countries in an attempt to reduce the number of pneumonia-related deaths.^5^ ^,^ ^6^ ^,^ ^7^These guidelines rely on simple clinical signs and consist of three steps:

1. identifying children in whom pneumonia should be investigated,
2. identifying pneumonia cases, and
3. administering appropriate antibiotic treatment.^8^

In addition, in 2012, WHO provided new recommendations for the use of first-line antibiotics and reset the classification of pneumonia severity.^7^ The distinction between the previously-defined “pneumonia” (rapid breathing) and “severe pneumonia” (chest indrawing) was no longer considered sufficient. The new classification, also comprised of two categories, was modified to include the appropriate therapy: “pneumonia” with fast breathing and/or chest indrawing, which requires home therapy with oral amoxicillin; and “severe pneumonia”, referring to pneumonia with any general danger sign, which requires hospitalization and intravenous therapy.^8^ Nevertheless, the initial step of identifying children who should receive antibiotics or undergo chest radiography is based on clinical predictors.^9^

According to WHO, raised respiratory rate (also referred to as tachypnea) as determined by visual inspection is the most expressive clinical sign of pneumonia in children presenting with cough or breathing difficulty.^10^ ^,^ ^11^ Shann et al.^11^ proposed that intercostal retractions are a major sign of severe pneumonia in children and also a warning for immediate hospital referral. The authors suggested that isolated tachypnea, even with respiratory rate greater than 50 breaths/minute, is a reliable sign that antibiotics should be prescribed for home use in cases of pneumonia without other signs of severity. 

For decades, tachypnea has been one of the most relevant clinical signs of pneumonia, as children presenting with this condition are more likely to have pneumonia than those without it.^12^ Indeed, the recognition of signs such as tachypnea by parents should be the first step in detection of children at risk for pneumonia. In the developing world, these simple clinical criteria can be used to identify more than 80% of children who require antibiotic therapy for bacterial pneumonia.^5^ ^,^ ^13^ Consequently, empowering parents/caregivers to recognize signs and symptoms may facilitate early and appropriate treatment, thereby helping reduce child mortality.

Because caregivers are often the first to notice respiratory signs and symptoms of an acute respiratory infection (ARI) in children, the present study sought to verify their knowledge and perceptions regarding severity or early warning signs of ARI, including the recognition of tachypnea, and the need to seek emergency medical assistance. Additionally, based on this information, this study aimed to teach parents/guardians to successfully recognize early warning signs and to seek medical assistance when appropriate.

## METHOD

Data were obtained from a cross-sectional study of children and adolescents under 15 years of age who were consecutively referred to the emergency room at the University Hospital of the Medical College of Jundiai (HU-FMJ), São Paulo, Brazil, from August 2011 to May 2012 during weekdays. HU-FMJ is a maternal and pediatric reference center with approximately 120,000 pediatric emergency entries annually. The catchment area of Jundiaí micro-region includes approximately 650,000 inhabitants, according to national census data from 2010.^14^ The only exclusion criterion was refusal to participate in the study.

**Figure 1:**
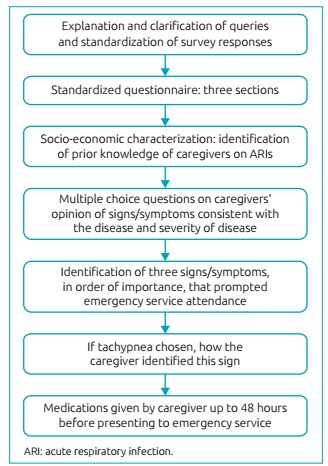
Standardized questionnaire used to identify caregivers’ socioeconomic characteristics, children’s comorbidities, and caregivers’ knowledge about ARI characteristics.

Data were collected from children’s parents or guardians using a previously tested standardized questionnaire, composed of multiple-choice and open questions using terms suitable for the lay audience. A trained investigator applied the questionnaires to ensure that respondents understood the questions and to clarify possible misinterpretations (Figure 1).

The questionnaire was divided into three sections: the first had questions about parents/guardians’ socioeconomic status and was aimed to evaluate their general knowledge about ARI. The second section addressed the child’s previous history of respiratory diseases (with ID in list). Finally, the third section pertained to the current infection and the caregiver’s perception of signs/symptoms considered worrisome, which led them to seek medical care.

Caregivers were presented a list from which they could select one or more common respiratory diseases that they considered to be the reason for seeking care at an emergency room (pneumonia, flu syndrome, colds, asthma, bronchitis, pharyngitis/tonsillitis or other diseases). Then, they were shown a second list, from which they could select one or more symptoms that they considered early warning signs of severe respiratory disease: rapid breathing, shortness of breath, coughing, runny nose, nasal congestion, fever, weakness, clear or yellowish/greenish phlegm, wheezing, painful breathing, difficulty breathing, purplish lips and fingertips, refusal of food and drink. Any queries about the symptoms were clarified. In addition, two open-ended questions were posed: one sought the opinion of caregivers about which respiratory disease the child was presently experiencing; the other asked caregivers to cite three of the previously identified symptoms that would be most worrisome in children, justifying a visit to the emergency room. They were also asked whether the children had received any medications over the previous 48 hours, so one could determine the possibility of disease severity masking with the use of common over-the-counter medications.

A multivariate analysis was performed to identify the variables correlated with tachypnea. This symptom is considered the most important early warning sign of severe ARI. To classify socioeconomic status, the Economic Classification Criteria (2011/2012) were used, as recommended by the Brazilian Research Association (ABEP). Such criteria estimate the purchasing power of urban individuals and families (http://www.abep.org/criterioBrasil.aspx). The system is based on two major axes: possession of items of consumption and educational level of the household head. Each item is assigned a score, and the total ranks families into eight economic classes (A1, A2, B1, B2, C1, C2, D and E). A1 economic class is reflected by a total of 42-46 points; A2 (35- 1); B1 (29-34); B2 (23-28); C1 (18-22); C2 (14-17); D (8-13) and E (0-7 points). The value corresponds to an average gross monthly family income of $5006.66 USD for A1; $2832.77 for B1; $1434.49 for B2; $910.74 for C1; $619.95 for C2 and $419.43 for DE class.

The variables are described as n (percentage). Chi-square and Student’s t-tests were performed to determine which variables contributed with the caregivers’ recognition of an acute respiratory disease picture. Double-entry of data was carried out and analysis was done using SAS-PC^®^ version 9.1 (SAS Institute, Inc., Cary, NC, USA). This research was conducted in accordance with ethical standards, the international guidelines established by the Declaration of Helsinki of 2013, and Resolution 466/2012 of the Brazilian Ministry of Health. Additionally, the Institutional Review Board approved the project.

## RESULTS

Interviews with 499 parents/guardians were conducted for the purpose of this study, and no respondent was excluded. Most caregivers were females (87.4 vs. 12.6% males), sorted in the following age groups: 15-30 years (55.7%), 31-50 years (39.8%), and 51-74 years (4.4%).

A similar number of male and female children were included in the sample (50.1% females vs. 49.9 males) and they were arranged in the following age groups: 0-2 years (48.1%), 3-4 years (19%), 5-10 years (22.6%), and 11-15 years (10.2%). Most of them (72.2%) did not have any previous diseases.

The caregivers’ socioeconomic status and children’s previous disease types are shown in Table 1. Parents/guardians cited flu syndrome (n=392, 78.6%), common cold (n=396, 73.9%), pharyngitis (n=320, 64.1%), bronchitis (n=307, 61.5%), pneumonia (n=272, 54.5%) and asthma (n=217, 43.5%) as the most common respiratory diseases in childhood. Allergic rhinitis was the most frequently cited as “other” respiratory disease (n=13, 2.6%).

**Table 1:** Baseline characteristics of the 499 caregivers and children.

	Patients (n)	Percentage (%)
Caregivers’ Socioeconomic Status		
A1	0	0
A2	3	0.6
B1	23	4.6
B2	94	18.8
C	329	65.9
D	49	9.8
E	1	0.2
Children’s Previous Disease Type		
Bronchitis	43	37.7
Rhinitis	21	18.4
Asthma	19	16.7
Allergies	10	8.8
Other	21	18.4

Table 25 lists the major symptoms that led parents/guardians to seek medical assistance for their children and caregivers’ perceptions regarding the signs of ARI severity. Almost all respondents cited fever as the most important sign of ARI severity (99.6%), followed by dyspnea, wheezing, difficulty breathing and weakness. Tachypnea was identified as a sign of ARI severity by 78.6% of respondents. The least cited symptoms/signs were clear phlegm and cyanosis. Table 2 also lists the symptoms/signs that parents/guardians reported to make them recognize respiratory disease in their children. Most of them acknowledged tachypnea and rapid breathing (41.7%) or fatigue (12.2%) as signs of ARI. Other items were reported by less than 5% of respondents. The symptoms considered the most worrisome by caregivers, leading them to seek emergency assistance, were dyspnea (n=147, 29.5%), tachypnea (n=119, 23.8%), and fever (n=107, 21.4%) (Table 2).

**Table 2:** Identification of signs and symptoms reported in questionnaires by respondents.

Major symptoms reported by respondents justifying their child’s need for medical care		
Symptom*	Respondents (n)	Percentage (%)
Fever	170	34.1
Coughing	79	15.8
Sore throat	24	4.9
Vomiting	23	4.6
Stomach ache	21	4.2
Shortness of breath	19	3.8
Earache	14	2.8
Painful urination	12	2.4
Wheezing	10	2
Yellow phlegm	8	1.6
Chest congestion	7	1.4
**Perceived signs and symptoms of severe respiratory disease**		
**Symptom**	**Respondents (n)**	**Percentage (%)**
Fever	497	99.6
Dyspnea	456	91.4
Wheezing	431	86.4
Difficulty breathing	425	85.2
Weakness	400	80.2
Coughing	398	79.8
Tachypnea	392	78.6
Refusal of food and drink	378	75.8
Nasal congestion	356	71.3
Runny nose	341	68.3
Pain when breathing	328	65.7
Yellow phlegm	322	64.5
Clear phlegm	227	45.5
Cyanosis	210	42.1
**Most worrisome symptoms cited by parents/caregivers which led them to seek emergency treatment**		
**Symptom**	**Patients (n)**	**Percentage (%)**
First symptom		
Dyspnea	147	29.5
Tachypnea	119	23.8
Fever	107	21.4
Second symptom		
Dyspnea	100	20
Fever	95	19
Tachypnea	48	9.6
Third symptom		
Fever	71	14.2
Difficulty breathing	67	13.4
Wheezing	53	10.6

*Note: other symptoms were cited by less than 1% of respondents.

The drugs most commonly given to their children 48 hours prior to seeking care were: antipyretics (n=207, 42%), with dipyrone for 125 (25,0%), paracetamol for 66 (13.2%), ibuprofen for 16 (3.6%), and aspirin for 1 of them (0.2%). Antipyretics were followed by antibiotics, administered to 17 children (3.4%). Other medications included: fenoterol hydrobromide (n=16, 3.2%), beclomethasone dipropionate (n=7,1.4%), and loratadine (n=5, 1.0%). 

The child’s history of respiratory disease (p=0.002), as well as the presence of old-age (p=0.010) and married caregiver (p=0.014), were significantly associated with early detection of tachypnea (Table 3).

**Table 3:** Variables significantly associated with tachypnea, considered the most worrisome sign of ARI.

	OR	95%CI	p-value
Children’s previous disease (yes)	2.11	1.32-3.37	0.002
Caregiver’s age (years)	0.97	0.95-0.99	0.010
Caregiver’s marital status (married)	1.75	1.12-2.73	0.014

OR: odds ratio; 95%CI: 95% confidence interval for the OR.

## DISCUSSION

Any intervention that reduces the number of deaths caused by pneumonia in children under five years is of great importance to public health^5^ and should be given consideration in any ARI prevention program. The ideal diagnostic marker of bacterial pneumonia should be accurate, minimally invasive, and readily available.^4^ Depending on the age of the patient, tachypnea might fulfil the criteria of an ideal marker; however, many variables, such as fever, wheezing, dehydration, and anemia can affect the ability of a caregiver to detect tachypnea.

The parents/guardians who participated in our study were chiefly adolescents and young women of low socioeconomic status (class C), and most of the patients were younger than two years old, constituting the most-at-risk group for pneumonia. Parents/guardians cited viral diseases as the most common causes of childhood ARI; an important complication, pneumonia ranked the fifth most common cause. This finding suggests limited knowledge about serious complications of ARI and indicates inappropriate use of healthcare services.

Increased breathing effort is an early warning sign and precedes changes in blood gases. Therefore, recognizing respiratory distress in children is utterly important for ARI early detection and treatment, in order to avoid complications and more invasive treatments.^15^ ^,^ ^16^ Although caregivers stated that they understood changes in breathing pattern such as dyspnea and difficulty in breathing as important early warning signs, these were not the main reasons they sought emergency services. There may still be a lack of understanding of severe respiratory disease signs.

Despite knowing that ARI has viral etiology and is usually a self-limiting disease, parents/guardians sought emergency care even with mild symptoms - also bearing in mind that only 22.8% of children had medical history, the most frequent case being hyper-reactivity of the lower airways (ICD codes J40-J47). This practice typically leads to overcrowding of emergency services, inadequate care, and increased costs due to treatment. In a large study conducted in the United Kingdom, McHale et al.^17^ found that 11.7% of emergency visits were rated as inadequate, with the highest rates of inadequacy occurring among 1- to 2-year-olds and patients living in disadvantaged areas. The costs of primary care associated with coughing in UK were estimated in £31 million, most of which was spent on medical staff.^18^

This study shows that fever was the primary reason to seek emergency care. This may be attributed to the anxiety of parents/guardians^19^ ^,^ ^20^ and the lack of primary care resources available. Fever is not a reliable sign of pneumonia in children because it also occurs in other childhood diseases^21^ and can markedly interfere with respiratory rate. A study conducted in The Gambia with children presenting with cough showed that the average respiratory rate increased by 2.5 breaths per minute for each 1°C higher in body temperature.^22^

Parents/guardians did not cite tachypnea as a reason for seeking emergency care for their children. Tachypnea requires immediate treatment; thus, all caregivers should be able to identify it to avoid unfavorable outcomes.^2^ ^,^ ^23^ A study that examined ARI-related deaths in developing countries found that 50% of all caregivers did not recognize signs of severity before death occurrence.^24^

Recognizing tachypnea may be difficult because caregivers do not frequently understand the biomedical definitions of diseases.^25^ In our study, most parents/guardians had a sense of the importance of tachypnea and how to evaluate it. A multivariate analysis showed that the recognition of this sign was significantly associated with children’s history of respiratory disease and with caregiver’s age and marital status. A total of 208 (41.7%) caregivers reported recognizing tachypnea by the acceleration of respiratory movements. Others reported noting that their children seemed tired or had an accelerated heart rate, confirming that they had not received previous information on tachypnea. Therefore, interventions that train parents/guardians and healthcare professionals to recognize the signs of tachypnea and the appropriate time to seek medical care are needed.^26^ Community intervention studies have shown a significant increase in care and a 32% reduction in mortality by pneumonia following the education of caregivers about ARI signs and symptoms.^27^

The strengths of this study, particularly compared to similar studies, are:

1. This was a consecutive series of emergency room admissions of a population from a defined acre area.
2. Data about hospital care were based on interviews with parents/caregivers, and we included children treated at the hospital regardless of their hospitalization status.
3. HU-FMJ is the only referral center for area that includes neighboring towns with approximately 900,000 inhabitants.

This study also had some limitations. The assessment of healthcare knowledge levels in emergency services might have lacked accuracy because parents/guardians were concerned with their children’s conditions and could not have been able to fully focus on completing the questionnaire. The majority of the population treated at the hospital lived in Jundiaí, the city with the highest socioeconomic status in the micro-region. Therefore, participants may not have accurately reflected the entire population, which includes poorer regions and more remote rural areas. Also, as the medical reports containing the full clinical assessment of these children were unavailable, it was not possible to compare the caregivers’ assessment of severity with that of the medical staff.

Although parents/guardians were able to intuitively recognize that rapid breathing is a sign of respiratory illness severity, they did not consider it sufficiently important to seek medical attention. Caregivers were also unable to acknowledge the severity of certain clinical signs such as fever. Difficulties in recognition of tachypnea documented in this study indicate that disease severity is not as effectively assessed at primary care visits as it is on presentation at hospital emergency departments. Grant et al.^28^ obtained similar findings when it comes to antibiotic administration by primary care facilities for children subsequently hospitalized with community-acquired pneumonia. Opportunities to identify tachypnea are commonly missed and can cause failure or delay in pneumonia diagnosis. The early management of pneumonia in children reduces mortality substantially.^5^

A lack of understanding of signs and symptoms that require monitoring in a child with suspected acute lower respiratory tract infection (LRTI) prompts parents/guardians to inappropriately seek medical care. In most cases, caregivers seek treatment after the patient’s condition has already deteriorated, requiring rescue intervention and occasionally invasive treatments that might not be effective. Thus, adequate guidance regarding these clinical manifestations, particularly tachypnea and intercostal retractions, must be implemented at all healthcare levels to reduce the number of preventable hospitalizations and deaths. In this context, broad dissemination of appropriate warnings to the general population, particularly to less-privileged strata, is desirable.

Recently, Rambaud-Althaus et al. reported that no individual clinical feature is sufficient for accurately diagnosing pneumonia and that using a combination of clinical features in decision-making might improve diagnostic performance; moreover, the authors reported that adding new point-of-care tests for diagnosis of bacterial pneumonia would help to reach an acceptable level of accuracy.^29^ Also, their survey points out the unrestrained use of analgesics/antipyretics by caregivers as possibly associated with symptomatic treatment of pain and fever. Pereira et al.^30^ reported identical findings in a large study addressing self-medication in children and adolescents. The same situation was detected with systemic antibiotics. This is an important fact that can contribute with adverse effects and development of bacterial resistance.

Based on this evidence, recognition of respiratory system changes related to ARI by parents/caregivers may contribute to early diagnosis and treatment, significantly reducing infant mortality rate by pneumonia. Tachypnea is rarely recognized by caregivers, and this finding reinforces the need for public policies of health prevention and promotion at all levels, including pediatric emergency services.
